# Generalized cost-effectiveness analysis of a package of interventions to reduce cardiovascular disease in Buenos Aires, Argentina

**DOI:** 10.1186/1478-7547-7-10

**Published:** 2009-05-06

**Authors:** Adolfo Rubinstein, Sebastián García Martí, Alberto Souto, Daniel Ferrante, Federico Augustovski

**Affiliations:** 1 IECS, Institute for Clinical Effectiveness and Health Policy, Buenos Aires, Argentina; 2 Division of Family and Community Medicine, Hospital Italiano de Buenos Aires, Argentina

## Abstract

**Background:**

Chronic diseases, represented mainly by cardiovascular disease (CVD) and cancer, are increasing in developing countries and account for 53% of chronic diseases in Argentina. There is strong evidence that a reduction of 50% of the deaths due to CVD can be attributed to a reduction in smoking, hypertension and hypercholesterolemia. Generalized cost-effectiveness analysis (GCE) is a methodology designed by WHO to inform decision makers about the extent to which current or new interventions represent an efficient use of resources. We aimed to use GCE analysis to identify the most efficient interventions to decrease CVD.

**Methods:**

Six individual interventions (treatment of hypertension, hypercholesterolemia, smoking cessation and combined clinical strategies to reduce the 10 year CVD Risk) and two population-based interventions (cooperation between government, consumer associations and bakery chambers to reduce salt in bread, and mass education strategies to reduce hypertension, hypercholesterolemia and obesity) were selected for analysis. Estimates of effectiveness were entered into age and sex specific models to predict their impact in terms of age-weighted and discounted DALYs saved (disability-adjusted life years). To translate the age- and sex-adjusted incidence of CVD events into health changes, we used risk model software developed by WHO (PopMod). Costs of services were measured in Argentine pesos, and discounted at an annual rate of 3%. Different budgetary impact scenarios were explored.

**Results:**

The average cost-effectiveness ratio in argentine pesos (ARS$) per DALY for the different interventions were: (i) less salt in bread $151; (ii) mass media campaign $547; (iii) combination drug therapy provided to subjects with a 20%, 10% and 5% global CVD risk, $3,599, $4,113 and $4,533, respectively; (iv) high blood pressure (HBP) lowering therapy $7,716; (v) tobacco cessation with bupropion $ 33,563; and (iv) high-cholesterol lowering therapy with statins $ 70,994.

**Conclusion:**

Against a threshold of average per capita income in Argentina, the two selected population-based interventions (lowering salt intake and health education through mass-media campaigns) plus the modified polypill strategy targeting people with a 20% or greater risk were cost-effective. Use of this methodology in developing countries can make resource-allocation decisions less intuitive and more driven by evidence.

## Background

Chronic diseases – mainly CVD, cancer, chronic respiratory diseases and diabetes – were estimated to have caused over 60% of all deaths globally in 2005, of which more than 80% occurred in low and middle-income countries; almost half of these deaths will occur in people younger than 70 years old (compared to only 27% from this age group in high-income countries). It has been projected that by 2015, 41 million people will die from chronic diseases if there are no concerted efforts in prevention and treatment.[1]

In Argentina, chronic diseases likewise account for more than 50% of the overall morbidity and mortality. From a total of 285,941 deaths in 2004, CVD accounted for 93,972 deaths, with a coronary heart disease age-adjusted incidence of 360/100000 in men and 80/100000 in women and a stroke age adjusted incidence of 120/100000 in men and 75/100000 in women, a pattern similarly found in other upper-middle income countries. [2] In common with many other Latin American countries, Argentina falls into an intermediate mortality group where the main risk factors for disease are hypertension, an elevated body mass index, alcohol abuse and smoking. [3] Primary data describing the prevalence and distribution of cardiovascular risk factors in the city of Buenos Aires has recently been obtained through two different population-based sources: the 2004 Ministry of Health National Risk Factor Survey [4]; and the Cardiovascular Risk Factor Multiple Evaluation in Latin America (CARMELA) [5]. The former surveyed a probabilistic sample of almost 50,000 households from all Argentine districts to detect risk factors, and the latter assessed the prevalence of cardiovascular risk factors and common carotid intima-media wall thickness distributions in a probabilistic sample of individuals living in 7 cities in Latin American, including Buenos Aires.

The WHO recently addressed the importance of chronic disease prevention as a neglected health issue in low- and middle-income countries; achievement of the global goal to reduce chronic disease death rates by 2% every year would avert 36 million deaths between 2005 and 2015. [6,7] Achieving this target would also save almost 10% of the expected loss in national income in these settings[8] There is strong evidence that a 50% reduction in cardiovascular deaths can be attributable to the reduction of just three modifiable risk factors, namely tobacco consumption, high blood pressure and elevated cholesterol [8] Moreover, at least 75% of CVD can be explained by more proximal risk factors like unhealthy diet, low physical activity and tobacco consumption.

Most CVDs are preventable and there is evidence that supports the effectiveness of interventions to reduce the burden of CVD through strategies that reduce risk factors. Unfortunately, strategies to manage cardiovascular conditions have been largely developed for high-income countries which may not be affordable to most of the developing world. [9,10] The health resource allocation decision process is usually empirical and driven by political, social or financial issues. The utilization of scientific evidence related to the economic impact of the interventions to set health priorities and define public coverage policies is uncommon in developing countries. Although there has been widespread recognition of the benefit of cost-effectiveness evaluation to inform national health system priority setting, its potential has not been realized in the vast majority of countries. We identified in a qualitative study conducted with Argentine healthcare decision makers that even though economic considerations to prioritize resource allocation were increasingly accepted, and boosted after the recent financial crisis, the use and application of economic evaluations was very poor and restricted to a limited handful of cases. [11]

Cost-effective interventions to prevent CVD in developing countries do exist, but have not been widely applied. Specifically, population and community-based interventions appear to be highly cost-effective when they reach large populations, address high mortality and morbidity diseases, and include multi-level integrated efforts. Interventions targeting individuals, especially high cardiovascular risk subjects, are also cost-effective but usually require clinical involvement and more resources. Moreover, recent studies have consistently shown the cost-effectiveness of interventions that lower the burden of CVD in developing countries. [12,13]

Different types of cost-effectiveness analyses are available to evaluate packages of interventions in terms of their cost-effectiveness. Generalized Cost-Effectiveness (GCE) analysis is a methodology designed by the WHO to evaluate the current and potential coverage of health interventions in order to improve allocative efficiency and to facilitate policy makers' ability to make informed decisions about health resource allocation. [14] Following a request in 2005 by the Secretary of Health of the city of Buenos Aires, the aim of this study is to develop a decision making framework based on GCE methodology to help policy makers identify the most cost-effective interventions to diminish the burden of CVD in Buenos Aires, Argentina.

## Methods

### Study population and perspective

Buenos Aires is the capital city of Argentina. According to the last national census (2001) it has a population of 3,053,030 of which, 26.5% rely on public health services alone while the majority has supplementary health care coverage from social security or private health insurance plans. The public health infrastructure of Buenos Aires is composed of 66 primary health care centers, 33 hospitals and 3,200 primary health care professionals, 50% of them being primary care physicians.

We incorporated the perspective of the public sector of the City of Buenos Aires. Because health care for the uninsured is a primary responsibility of the local government through the public healthcare network, this study aimed to reflect the cost-effectiveness and budgetary impact of providing individual interventions to the uninsured population specifically. In contrast, the population-based interventions were calculated as being delivered to the whole population regardless of their insurance status.

### Definition and selection of interventions

A total of eight population-based and individual primary prevention interventions were selected to evaluate their impact over a 10 year period. The interventions were selected based on their common use as primary preventions of CVD and evidence of efficacy and effectiveness. The interventions had also been already selected in a previous landmark study [12].

#### Population-based interventions

1) Intervention to Reduce Salt Intake: Program involved the cooperation between Government, consumer associations and the Bakery Chambers to reduce 1 gram of salt per 100 grams of bread. Argentina has a consumption of 12 grams of salt per day, 3.4 grams coming from bread. Local experiences showed that it is possible to reduce the amount of salt in bread without being detected as less palatable. At present, there is a pilot training program implemented in selected cities in Argentina to make bakers reduce salt in bread by using special salt dispensers. [15] This intervention could imply a population-wide reduction of 1.33 mmHg of systolic blood pressure per person (SBP) and 1% of the population attributable risk (PAR) of coronary heart disease (CHD) and stroke. [16]

2) Public Education through mass media: Health education through broadcast and print media promoting healthy habits, low fat diet and low salt consumption. A meta-analysis of community-based interventions through mass media campaign, showed a decrease of 1.83 mmHg of SBP in SBP and a 0.02 mm/l in cholesterol (t), implying a reduction of 2% of the PAR of CHD and stroke. [17]

#### Individual Interventions

Six interventions were clinical interventions targeted towards the uninsured (26.5% of the population of Buenos Aires).

1) Individual treatment of high blood pressure: Intervention involved lifestyle change promotion and pharmacological therapy to achieve blood pressure control (SBP/DBP less than 140/90). In order to conduct the GCE analysis we assumed that 40% of the population would take one drug, 40% at least two drugs and 20% three or more drugs. The drugs and daily doses evaluated were hydrochlorothiazide (25 mg), atenolol (50 mg) and enalapril (10 mg), the same efficacy for each drug category was also assumed Analysis indicated that this interventions, with a 50% rate of disease detection and drug compliance indicated by the Canadian Hypertension Guidelines, would reduce PAR of CVD and stroke by 8%.[18]

2) Individual treatment of high cholesterol: Promotion of low-cholesterol diet and statin use (atorvastine 10 mg) to achieve a Cholesterol (t) target of less than 240 mg/dl, (6.2 mm/l) provides an estimated reduction of 8% of the PAR of CHD and stroke [19] a 50% detection and drug compliance rate according to ATP III. [20]

3) Individual tobacco cessation therapy: Drug therapy with bupropion for a 2-month period (300 mg per day) results in an estimated reduction of 4% of the PAR of CHD and stroke. [21] According to a recent national survey of tobacco prevalence [22] only 13% of total smokers in Argentina were willing to quit smoking and therefore are considered the target population of the intervention.

4) Treatment based on a population absolute risk approach (modified polypill strategy): Pharmacological therapy with thiazides 25 mg, enalapril 10 mg, atorvastatin 10 mg and aspirin 100 mg prescribed to people with an estimated combined risk of a cardiovascular event over the next decade above a given threshold (>5%, >10% or >20%) implying a reduction of PAR of CHD and stroke of 15%, 40% and 60% for each risk group, respectively. [23] We assumed a 50% compliance rate in those with a 10 year risk of 5% and 10%, and 80% compliance in those with a 20% risk. The prevalence and values of high blood pressure, high cholesterol and smoking in Buenos Aires were obtained from recent study estimates [4,5]. The number of subjects in each risk strata was estimated by using the beta coefficients from the Framingham Heart Study. [24]

### Modeling of intervention effects

Our analytic model was based on the WHO-CHOICE methodology. This approach entails lifting the constraints of the current mix of interventions, using a null scenario of no costs and no interventions as a starting point to estimate allocative – as opposed to productive/technical – efficiency in the health sector.[14] The null scenario was calculated taking into account that the only individual intervention of those selected, currently provided by the Secretary of Health to the uninsured, is hypertensive therapy, while statins and bupropion are not yet covered by the public health care system.

In order to estimate the reduction in disease burden related to the reduction of CVD, we needed a model to predict the burden associated with specific diseases or risk factors to develop disease. In table 1 we show base-case estimates of the relative risks (RR) of CHD and stroke and its respective sources. We used Population Attributable Risk (PAR) as a measure of impact of each risk factor on the incidence of CHD and Stroke where

**Table 1:** Relative Risks for Coronary Heart Disease (CHD) and Stroke for different conditions

Risk factor	CHD RR	Reference	Stroke RR	Reference
Hypertension	1.91	[33]	4	[34]
Tobacco use	1.68	[24]	2	[35]
Hypercholesterolemia	2.5		1	[36]
More than 5% CVD global risk *	8.84	[4,5]	8.84	[4]
More than 10% CVD global risk*	13.12		13.12	
More than 20% CVD global risk*	18.8		18.8	

CVD: Cardiovascular disease.

* Local Population-based age and sex specific prevalence of cardiovascular risk factors and its observed distribution were used. Then, we derived global CVD risk using Framingham Risk Equation (25) to estimate CVD risk in each stratum (more than 5%, more an 10% and more than 20%)



Then, with the estimate of the Relative Risk (RR) of the intervention and the PAR, we calculated the percent PAR reduction of CHD and Stroke as a consequence of the intervention as follows:



Finally, the model translated these changes in cardiovascular risk events specific for age and sex (Δ PAR before and after the intervention) into changes in population health, quantified by number of DALYs averted. Effect sizes and joint effect of interventions used in the analysis were based on systematic reviews of randomized trials and meta-analysis, when possible. Intervention effects with their corresponding relative risks (RR) estimates are shown in Table 2.

**Table 2:** Relative risks of proposed interventions

**Interventions**	**RR (point estimate used in the model)**	**Reference source**
**Population-based**		
Health education through Mass Media	0.98 (1)	[17]
Reduction of salt in bread through voluntary agreement	0.99 (2)	[16]
**Individual (clinical)**		
Pharmacological Treatment of High Blood Pressure	0.82	[37]
Pharmacological Treatment of High Cholesterol	0.95	[19]
		
Tobacco cessation therapy with bupropion	0.8	[21]
Combined therapy for patients with> 5% global risk	0.12 (3)	[23]
Combined therapy to > 10% global risk		
Combined therapy to > 20% global risk		

(1) The translation of reduction in mmHg and cholesterol mg reported by the intervention to relative risk was done using Framingham Risk Equations from Wilson et al, Circulation 1998 [24].

(2) Average population bread consumption was estimated from the Agricultural and Food Secretariat. The average amount of salt in bread was obtained from a survey from the National Institute of Industrial Technology (INTI 2005). Then, we applied the same equation as in (1) to transform mmHg decreases in relative risk reduction

(3) We subtracted the folic acid effect from the polypill paper due to its lack of consistent evidence of efficacy

Population health effects due to the interventions were modeled simulating population specific for age and sex with the observed baseline values of cardiovascular risk and the observed distribution of risk factors drawn from local data [4,5]

To translate changes in the risk of age and sex specific CVD events into changes in population health quantified in terms of DALYs, we used a standard multi-state modeling tool develop by the WHO-CHOICE group, PopMod. In this model, health effects are estimated by tracing what would happen to each age and sex cohort of a given population over 100 years with and without the intervention.[14] PoPMod is a four-state population model simulating the evolution of a population split into 4 different health states: people who have one condition (i.e. CHD), people who have another condition (i.e. Stroke) people who have both conditions (i.e. CHD and Stroke), and people who have none of the above but are at risk. Births and deaths are also included and transition rates such as incidence, remission, and mortality, govern movements between states.

Transitional probabilities and disability weights of CVD and stroke were obtained from WHO-Choice CVD template that is based on Framingham data. The entire population is subject to background mortality and morbidity, which are assumed to be independent of the CVD states explicitly modeled. In summary, this model projects the effect of interventions on the aggregate healthy years of life lived by a population, combining incidence, prevalence, and mortality and estimates of disease severity with information on intervention coverage and effectiveness. [25].

### Intervention Costs

Costs included program-level expenses associated with management of the interventions (i.e., administration, training and information dissemination by multiple media sources) and patient-level costs (i.e. primary care visits, ancillary tests and drugs). Potential cost-savings related to an event prevented by an intervention were not incorporated because the counterfactual or comparator situation is one without intervention (null scenario). The quantities of each input required were assessed and multiplied by the unit price of each input for the 10 year-intervention implementation period. For each program cost the quantities of required inputs were identified from similar programs conducted in the City of Buenos Aires or from expert opinion where necessary. The quantity of patient-level resource inputs for each intervention (i.e. inpatient hospital days, doctor visits, tests, drugs) were identified from local or international published data where available or from expert opinion. Costs of drugs were calculated using a mix of blood pressure lowering drugs composed of 50% thiazides, 20% beta blockers, 20% ACE inhibitors and 10% calcium channels blockers, according to a recently published study.[26] Cost of blood pressure lowering drugs, atorvastatine and bupropion, other input costs and expense data, as well as other cost data, were extracted from the purchase database of the Health Ministry of Buenos Aires City Government and the Institute of Clinical Effectiveness and Health Policy Unit Costs database. A list of the costs and sources of the interventions and selected health events is depicted in Table 3.

**Table 3:** Interventions and related health events summary costs

**Event cost per hospital admission**	ARS $
Coronary Heart Disease	2,879
Stroke	1,682
	
**Total intervention cost per year**	
Health education through Mass Media^1^	634,069
Less salt in bread^2^	87,471
	
**Yearly cost per person**^3^	
Hypertension treatment	39.54
High Cholesterol treatment	70.19
Bupropion treatment for tobacco cessation	109.73
Modified Polypill strategy	92.71

1. Ten years duration of campaign, more intensive during the first two years and with periodic reinforcement over the ten-tear period.

2. Assuming 54 meeting of 30 bakers each (aprox. 800-600 bakers). These meetings will be carried out during the first two years with reinforcement meetings for years fifth and sixth and ninth and tenth.

3. Includes health center visits, drug and lab test costs.

Cost of blood pressure lowering drugs, atorvastatine and bupropion as well as other input costs and charges data were extracted from the purchase data of the Health Ministry of Buenos Aires City Government (available at  comprassalud, 2005). Other cost data were obtained from the Health Care Costs Database by the Institute of Clinical Effectiveness and Health Policy (accessed at  Base de Datos de Costos Sanitarios Argentinos).

Except when explicitly stated, additional costs (i.e. a program to identify high cardiovascular risk people), costs related to labor, equipment, capital, overhead or joint costs were regarded as existing, ongoing, or common to all interventions and therefore were excluded in the calculation. We excluded costs of accessing health interventions that would include the resources used by patients and their families to obtain an intervention (transport costs) as well as productivity gains or losses, as the study was conducted from a purchaser perspective. All costs were calculated in Argentine pesos for the year 2005 (ARS $3.01 = US $1 exchange rate on March, 2005). The discounting of long term costs were performed at a 3% rate.

### Calculating cost-effectiveness

Cost-effectiveness analysis generally considers the costs and effects of adding new interventions to current practice or the cost of replacing an existing intervention with another targeting the same condition. Here we evaluated the proposed interventions by first considering what would happen to the population health if they all ceased to be implemented today. This is the null or 'do nothing' scenario. Average cost-effectiveness ratios were then calculated for each intervention by combining the information on total costs with information on the total health effects in terms of DALYs averted. DALYs are age-weighted and discounted at 3% per annum. To estimate the financial impact of conducting the interventions at different budget scenarios, costs were then compared with their health gains to identify the most cost-effective set of interventions at different levels of resource availability.

### Sensitivity analysis

Selected one-way sensitivity analyses were undertaken to assess the effects of uncertainty in the assumptions on the baseline levels of risks and effect sizes of interventions. The analysis was conducted taking into account an uncertainty of 20% around the central estimate of each variable. In addition, an undiscounted scenario was considered for costs and DALYs, and a non age-weighted scenario was also analyzed for DALYs.

## Results

The prevalence of high blood pressure, high cholesterol and smoking, as well as the percentage of target population in each 10-year CV risk strata (>5%, >10%, and >20%) in Buenos Aires can be seen in Table 4. During 2004, there were 1,338 hospital admissions with a diagnosis of CHD and 977 with a diagnosis of stroke at an average cost per admission of ARS $2,879 and ARS $1,682 for CHD and stroke, respectively

**Table 4:** Prevalence of Cardiovascular risk factors and risk strata in Buenos Aires by gender (1)

**Prevalence**	**Males/Females (%)**
High Blood Pressure	40/26
High Cholesterol	22/22
Smoking	40/35
**Cardiovascular risk (2)**	**% of target population**
Over 5%	45
Over 10%	25
Over 20%	6

(1) Uninsured population covered by Buenos Aires city public healthcare network (approximately 800.000 persons)

(2) % of subjects in each cardiovascular risk strata were derived from Framingham equations using risk factor values obtained from population surveys of Buenos Aires, Argentina

**Figure 1:**
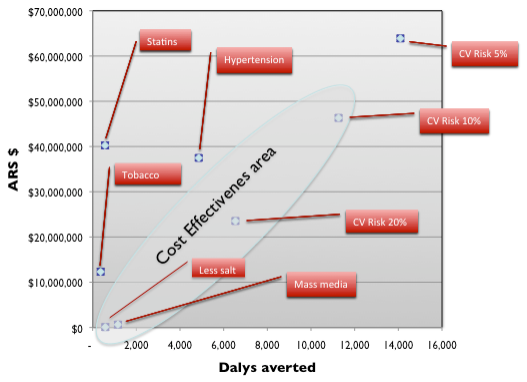


Table 5 gives the total annual costs and, total annual health effects in terms of DALYs averted (age-weighted and 3% discounted, and non-age weighted and undiscounted) and the average cost-effectiveness ratio for each of the 8 distinct interventions. Concerning specific interventions, the strategy of lowering salt intake in the population through reducing salt in bread was found to be the most cost-effective (ARS $151 per DALY averted), followed by health education through mass-media campaign (ARS $547 per DALY averted) and the modified polypill strategy. This pharmacologic approach to patients with an estimated combined risk of a cardiovascular event over the next decade above 20%, 10% or 5% showed a cost-effectiveness ratio of ARS $3,599, $4,113 and $4,533 per DALY averted. respectively. Because these interventions are mutually exclusive, only one of the three cut-off points can be selectedOn the other hand, interventions targeted at individual risk factors like high blood pressure control with antihypertensive drugs, treatment of high cholesterol with a statin and tobacco cessation therapy with bupropion, ranked lower than the previous three and were dominated, as shown in Figure 1. Lowering cholesterol with statins and tobacco cessation with bupropion were not found to be cost-effective, in part because statins and bupropion are much more expensive than HBP lowering drugs and also because as they are not currently covered, the government does not usually exert its purchasing power to get lower prices. In addition, and in accordance with local surveys as mentioned above, as long as we assumed that only 13% of the population of smokers would be willing to quit smoking each year and consequently start on a program, the population impact of tobacco cessation therapy was much smaller than expected.

**Table 5:** Costs, effects and cost-effectiveness of interventions analyzed

**Intervention**	**Total Cost per year (ARS$) (1)**	**DALY Age weighted, 3% discounted per year (2)**	**DALY No age-weight 3% discounted per year**	**DALY # age-weight, undiscounted per year**	**ARS$ (1)/DALY (2)**
**Less salt in bread**	$ 87,471	579	713	1,107	$ 151
**Mass media campaign**	$ 634,069	1,158	1,426	2,213	$ 547
**Combined therapy 20% global CV risk**	$ 23,533,467	6,539	8,033	12,468	$ 3,599
**Combined therapy 10% global CV risk**	$ 46,323,335	11,263	13,913	2,163	$ 4,113
**Combined therapy 5% global CV risk**	$ 63,893,600	14,095	17,409	2,706	$ 4,533
**HBP lowering therapy**	$ 37,478,853	4,857	5,919	9,185	$ 7,716
**Tobacco cessation therapy with bupropion**	$ 12,317,628	367	449	697	$ 33,563
**High-chol lowering with statins**	$ 40,253,626	567	712	1,087	$ 70,994

### Results of sensitivity analysis

In order to estimate the budget impact of the three most cost-effective interventions, we analyzed the Buenos Aires Health Ministry annual operative budget. Total annual budget for the year 2004 was ARS $1.2 billion. After reviewing all budget item lines, most of them were considered rigid in terms of the difficulties to shift money from one budget line to another (i.e. labor costs, facilities, equipment, capital, land, overhead, etc.). Nevertheless, we were able to identify a "flexible" budget of ARS $106 million, representing about 8% of the total budget. Subsequently two budget reallocation scenarios were built that considered a small part of the flexible budget to finance the interventions until the money was exhausted. The first scenario would use 10% of this flexible budget (ARS $10,600,000) while the second scenario would use 20% (ARS $21,300,000).

As shown in Table 6, the reallocation of 10% of the flexible budget to this selected intervention set could finance the two population-based interventions, namely less salt in bread through negotiations and regulations with the Bakery Association and a mass-media campaign to educate people on healthy habits, low fat diet and low salt consumption (averting 250 DALYs per 100,000 subjects over 10 years); the remaining 9 millions could be used to start financing programs to detect, diagnose and treat subjects with high cardiovascular risk, saving additional DALYs in this population. The reallocation of 20% of the budget (and assuming non-divisibility of clinical interventions) could almost quadruple DALYs averted, enabling the addition of the modified polypill strategy to subjects with an estimated cardiovascular risk above 20%. This last outcome would be reached using less than 3% of the total annual operative budget of the district.

**Table 6:** Purchasing options using flexible budget reallocation scenarios to finance interventions

**Budget per year ARS $**	**Interventions**	**Cost per year period** **(ARS $)**	**DALYs averted**	**DALYs averted per 100.00 population**
10.6 millions (10% of the budget)	• Less salt in bread • Mass media campaign	$ 721,540	1,737	250 Dalys
21.3 millions (20% of the budget)	• Mass media campaign • Less salt in bread • CV risk 20%	24.2 millions	7,118	1050 Dalys

For this table, we assumed non-divisibility of the potential programmes.

## Discussion

In the context of the escalating burden of chronic disease in developing countries, this study set out to provide local decision-makers with information comparing the relative costs and health effects of interventions for preventing CVD, and in so doing focus policy debate concerning the trade-offs or opportunity costs of financing one intervention over another. Establishing the cost-effectiveness of chronic disease interventions in developing country contexts is not straightforward, however, owing to the paucity of existing information and evidence in these more resource-constrained contexts, and also because there is no universally agreed threshold for considering the cost-effectiveness of an intervention to be 'too high' or 'right'. What is acceptable to health and finance decision-makers depends on the country context. The Disease Control Priorities Project (DCPP), has identified several chronic disease interventions as cost-effective at a cost of below $1,000 per DALY. [27] However, the affordability of interventions will vary significantly across countries, even among a group of interventions believed to be cost-effective in the global sense. Moreover, sensitivity analysis done as part of the CEA modeling for the DCPP showed that the cost-effectiveness of public education campaigns at the population level could be very good or far less favorable depending on how much it cost to reach people using a reasonable range of costs. In addition, even a very inexpensive intervention might not be worth implementing if it targets a chronic disease with low prevalence in a given country or region.

In an earlier analysis that formed the basis for the present exercise, Murray et al. [12] modeled selected population-based and individual health interventions to lower high blood pressure and high cholesterol in the epidemiological contexts of developing countries. The authors found that all interventions were highly cost-effective in the sub-region of the Americas to which Argentina belongs.

More recently, Asaria et al., assessed the financial costs and health effects of a voluntary reduction in the salt content of processed foods by manufacturers plus a mass media campaign to encourage dietary change in 23 selected low and middle income countries, including Argentina. They estimated that a 15% reduction in dietary salt intake in Argentina would save 60,000 lives over the period 2006–2015 at a cost of US$ 0.14 per capita (equivalent to ARS $1.2 million for a population the size of Buenos Aires (3 million).[28] The addition of individual-level interventions with a multi-drug regimen on the basis of opportunistic contact with the health service, by contrast, has been estimated at US$ 2.93 per capita in Argentina (around ARS 25 million for the population of Buenos Aires), but would save a further 50,000 lives over a 10-year period. [29]

As compared to these previous studies [12,28], our intervention to decrease salt intake, even though was aimed to reduce salt only in bread instead of reducing salt in all processed foods, had a similar cost-effectiveness ratio than the Asaria's study (ARS $ 151 per DALY vs. ARS $ 202 per DALY). In regards to the intervention oriented to reduce cardiovascular disease in subjects with different cardiovascular risks, health gains in DALYs averted in our study were remarkably similar to those reported by Murray et. al. for the same risk strata, although costs were much higher, Consequently, cost per DALY saved for each risk group was also higher in our study

In summary, the two selected population-based interventions (lowering salt intake and health education through mass-media campaigns) and the modified polypill strategy targeting people above 20% of cardiovascular risk in 10 years were very cost-effective according to the threshold adopted by WHO-CHOICE (an intervention that saves one DALY for less than three times gross national product (GNP) per capita is considered cost-effective, while one that saves a DALY for less than GNP per capita is deemed very cost-effecive). [30] As Argentina's GNP per person in 2005 was ARS $ 13,728 (US$ 4,470) [31], estimated CERs of each of these interventions fall well within the 'very cost-effective' category. However, as mentioned above, our results differ from those obtained in the aforementioned regional analysis: concerning salt, both our health impact and cost estimates are appreciably lower than those summarized above, partly because we only included a series of one-off meetings with bread makers, and also because we used a lower effect size. Concerning the modified polypill strategy, effects are also less than predicted by the regional models, but our cost estimates are considerably higher, which reflects the fact that key intervention resource inputs in Buenos Aires – including human resources, secondary care and drugs – are much more expensive than the regional average.

According to estimates from the Global Burden of Disease project, [32] the estimated burden in a single year due to HBP, high cholesterol and high body mass index (BMI) for America B countries add up to 5.47 million DALYs in men and 4.56 million in women, equivalent to 23,304 per 100,000 people over a 10-year period. Therefore, the implementation of these three integrated interventions (less salt in bread, mass media campaign to promote healthy lifestyles and drugs to prevent CVD in high-risk patients) in the Buenos Aires metropolitan area would save 1,200 DALYs per 100,000 individuals and would account for a 5% reduction in the burden of CVD. Moreover, considering that our hospital costs for acute myocardial infarction and stroke over 10 years were only in the range of ARS $2.1 million (discounted at a 3% annual rate), and the overall cost estimate for the selected interventions would be only 10% of the cost of acute events (ARS $205 million), these interventions would be cost-saving if the counterfactual scenario had been what the government is actually spending on the care of cardiovascular disease.

## Conclusion

Overall, evidence exists to conclude that there are important economic as well as clinical consequences of CVD, consequences that are not only important to the individual and his/her family but also to the economy at large. At the same time, there are severe gaps in the evidence that call for more research into the avoidable burden of CVD, and in particular for developing countries [33-37]. Taking into account the increasing burden of CVD in Argentina, ranking first over the last decades as a cause of mortality and morbidity, urgent action is needed to convince policy makers about the high yield of integrated interventions like the ones evaluated above.

Finally, although cost-effectiveness should be one of the key inputs for policy makers to make resource-allocation informed decisions, other criteria are usually taken into account, like equity, social acceptance, intuition, prior decisions, and healthcare infrastructure needed to "accommodate" new interventions, not mentioning less transparent criteria like personal interest and corruption. Nevertheless, this framework aims to help policy makers to make informed resource-allocation decisions when making choices to reduce the burden of CVD, especially in middle-income developing countries like Argentina.

## Competing interests

The authors declare that they have no competing interests.

## Authors' contributions

AR carried out and participated in the design of the model and analysis of the results. SGM carried out and participated in the design of the model and analysis of the results. AS provided the cost inputs of the model. DF carried out and participated in the design of the model providing epidemiological inputs. FA participate in the design of the study and helped to draft the manuscript.

## References

[B1] Mathers CD LA, Stein C, Fat DM, Rao C (2005). Deaths and disease burden by cause: global burden of disease estimates for 2001 by World Bank Country Groups. Working paper 18. Bethesda, MD: Disease Control Priorities Project.

[B2] Defunciones por causas, Argentina, 2004. Dirección de Estadísticas e Información. Ministerio de Salud de la Nación.

[B3] Lopez AD, Mathers CD, Ezzati M, Jamison DT, Murray CJ (2006). Global and regional burden of disease and risk factors, 2001: systematic analysis of population health data. Lancet.

[B4] (2006). Primera Encuesta Nacional de Factores de Riesgo. Primera Edición – Buenos Aires. Ministerio de Salud de la Nación.

[B5] Schargrodsky H, Hernandez-Hernandez R, Champagne BM, Silva H, Vinueza R, Silva Aycaguer LC, Touboul PJ, Boissonnet CP, Escobedo J, Pellegrini F, Macchia A, Wilson E, CARMELA Study Investigators (2008). CARMELA: assessment of cardiovascular risk in seven Latin American cities. Am J Med.

[B6] (2005). Preventing chronic diseases: a vital investment. WHO global report Geneva.

[B7] Strong K, Mathers C, Leeder S, Beaglehole R (2005). Preventing chronic diseases: how many lives can we save?. Lancet.

[B8] Abegunde DO, Mathers CD, Adam T, Ortegon M, Strong K (2007). The burden and costs of chronic diseases in low-income and middle-income countries. Lancet.

[B9] Murray CJLLA, Murray CJL, Lopez AD (1996). The Global Burden of Disease: a comprehensive assessment of mortality and disability from diseases, injuries, and risk factors in 1990 and projected to 2020. Cambridge.

[B10] World Health Organization (2002). The World Health Report: Reducing risks, Promoting Healthy Life.

[B11] Rubinstein A, Belizan M, Discacciati V (2007). Are economic evaluations and health technology assessments increasingly demanded in times of rationing health services? The case of the Argentine financial crisis. Int J Technol Assess Health Care.

[B12] Murray CJ, Lauer JA, Hutubessy RC, Niessen L, Tomijima N, Rodgers A, Lawes CM, Evans DB (2003). Effectiveness and costs of interventions to lower systolic blood pressure and cholesterol: a global and regional analysis on reduction of cardiovascular-disease risk. Lancet.

[B13] Gaziano TA, Opie LH, Weinstein MC (2006). Cardiovascular disease prevention with a multidrug regimen in the developing world: a cost-effectiveness analysis. Lancet.

[B14] Tan-Torres Edejer T, et al, World Health Organization (2003). Making choices in health: WHO Guide to Cost-Effectiveness Analysys.

[B15] (2005). Relevamiento del uso de sal en los productos de panaderías artesanales de la República Argentina e implementación de acciones de desarrollo, tecnológicas, de asistencia técnica y extensión con el objeto de bajar su utilización y consumo. Programa VIGI+A.

[B16] He FJ, MacGregor GA (2003). How far should salt intake be reduced?. Hypertension.

[B17] Sellers DE, Crawford SL, Bullock K, McKinlay JB (1997). Understanding the variability in the effectiveness of community heart health programs: a meta-analysis. Soc Sci Med.

[B18] (2004). The Canadian Hypertension Education Program Recommendations.

[B19] Law MR, Wald NJ, Rudnicka AR (2003). Quantifying effect of statins on low density lipoprotein cholesterol, ischaemic heart disease, and stroke: systematic review and meta-analysis. Bmj.

[B20] (2002). Detection, Evaluation and Treatment of High Blood Cholesterol in Adults. Adult Treatment Panel III.

[B21] Hughes J, Stead L, Lancaster T (2004). Antidepressants for smoking cessation. Cochrane Database Syst Rev.

[B22] (2005). Encuesta de Tabaquismo en grandes ciudades de Argentina – 2004. Ministerio de Salud y Ambiente de la Nación.

[B23] Wald NJ, Law MR (2003). A strategy to reduce cardiovascular disease by more than 80%. Bmj.

[B24] Wilson PW, D'Agostino RB, Levy D, Belanger AM, Silbershatz H, Kannel WB (1998). Prediction of coronary heart disease using risk factor categories. Circulation.

[B25] Lauer JA, Rohrich K, Wirth H, Charette C, Gribble S, Murray CJ (2003). PopMod: a longitudinal population model with two interacting disease states. Cost Eff Resour Alloc.

[B26] Gimpel N SV, Rubinstein A (2006). Quality Management of Hypertension in Primary Care: Do physicians treat blood pressure level or patients' risk?. Quality in Primary Care.

[B27] Jamison D (2006). 'Investing in health', in D Jamison et al., Disease Control Priorities in Developing Countries.

[B28] Asaria P, Chisholm D, Mathers C, Ezzati M, Beaglehole R (2007). Chronic disease prevention: health effects and financial costs of strategies to reduce salt intake and control tobacco use. Lancet.

[B29] Lim SS, Gaziano TA, Gakidou E, Reddy KS, Farzadfar F, Lozano R, Rodgers A (2007). Prevention of cardiovascular disease in high-risk individuals in low-income and middle-income countries: health effects and costs. Lancet.

[B30] Batussen RMAT, Tan Torres T (2002). Generalized cost-effectiveness analysis: a guide. Global Programme on Evidence for Health Policy.

[B31] http://siteresources.worldbank.org/DATASTATISTICS/Resources/GNIPC05.pdf.

[B32] Ezzati M, Lopez AD, Rodgers A, Hoorn S Vander, Murray CJ (2002). Selected major risk factors and global and regional burden of disease. Lancet.

[B33] Yusuf S, Hawken S, Ounpuu S, Dans T, Avezum A, Lanas F, McQueen M, Budaj A, Pais P, Varigos J, Lisheng L (2004). Effect of potentially modifiable risk factors associated with myocardial infarction in 52 countries (the INTERHEART study): case-control study. Lancet.

[B34] Kannel WB (2000). Risk stratification in hypertension: new insights from the Framingham Study. Am J Hypertens.

[B35] Brownson (1998). Chronic Disease Epidemiology and Control.

[B36] Bronner LL, Kanter DS, Manson JE (1995). Primary prevention of stroke. N Engl J Med.

[B37] Law MR, Wald NJ, Morris JK, Jordan RE (2003). Value of low dose combination treatment with blood pressure lowering drugs: analysis of 354 randomised trials. BMJ.

